# Political orientation, moral foundations, and COVID-19 social distancing

**DOI:** 10.1371/journal.pone.0267136

**Published:** 2022-06-24

**Authors:** Hammond Tarry, Valérie Vézina, Jacob Bailey, Leah Lopes

**Affiliations:** 1 Department of Psychology, Kwantlen Polytechnic University, Surrey, British Columbia, Canada; 2 Department of Political Science, Kwantlen Polytechnic University, Surrey, British Columbia, Canada; 3 Student Research Assistant, Kwantlen Polytechnic University, Surrey, British Columbia, Canada; Chinese Academy of Medical Sciences and Peking Union Medical College, CHINA

## Abstract

During the COVID-19 pandemic, governments have advocated numerous social distancing measures, and compliance with these has likely saved millions of lives globally. In an online sample drawn from the U.S. and Canada (N = 209), participants completed measures of political orientation, moral foundations, and COVID-19 social distancing attitudes and behaviours. A more left-wing political orientation, and greater endorsement of the individualizing moral foundations were significantly related to more positive social distancing attitudes, and greater self-reported compliance with relevant restrictions. A more right-wing political orientation, and greater endorsement of the binding and economic liberty foundations were associated with less positive attitudes and reduced compliance. In a series of mediation analyses, the relationships between political orientation and various social distancing measures were significantly mediated by variations in participants’ moral foundations, particularly their endorsement of economic liberty and the individualizing foundations. Further data indicated that the perceived persuasiveness of messages based on each moral foundation advocating for continued social distancing was significantly related to both participants’ moral values and their political orientation. Findings are discussed in terms of understanding politicized differences around social distancing as partly reflecting differential valuation of the moral foundations, and in creating effective public health messaging regarding compliance.

## Introduction

After the outbreak in late 2019 of a new coronavirus (SARS-CoV-2) and its associated respiratory illness (COVID-19), it rapidly progressed to a global pandemic by March 2020, which is ongoing at the time of writing in June 2021. To date, it has infected over 168 million people, and claimed over 3.5 million lives worldwide [1]. Its rapid spread led to dramatic governmental, behaviourally oriented measures to contain it around the world [2]. Even now, with the availability of effective vaccines [3], these measures remain a key component in overcoming the pandemic [4–6] in the face of more transmissible variants [7].

Despite national variations in the timing and severity of these behavioural measures [8], there have been common guidelines that many governments and health officials have advised or mandated. These include wearing face masks in public places and indoor venues [9], and more frequent, stringent handwashing [10]. Perhaps the most consistent guidelines have been that those who display symptoms of COVID-19 should self-isolate from others, while those without symptoms (who could still be infected due to asymptomatic spread) should engage in “social distancing” [11, 12].

Social distancing is the practice of staying at least 6 ft from other people and avoiding gathering in groups or crowded places [13]. It also involves reducing physical contact with people outside of the home, for example in social, work, or school settings [14]. Many governments have introduced such measures, including keeping a minimum distance from others, limiting socializing to that between household members, bans on large gatherings, stay-at-home measures, closures of non-essential businesses and services, and bans on non-essential travel [15–17].

The implementation of such measures has limited the spread of COVID-19, “flatten the curve” of case numbers during its multiple waves [14, 18], and reduced peak death rates, potentially saving millions of lives worldwide [19]. Given this, it is reasonable to assume that lack of adherence to these policies may have increased the lives lost and could continue to do so for as long as some restrictions are required [14].

Many studies in different countries have reported significant differences in individual adherence [20–22]. In turn, they have explored a variety of psychological factors that predict level of compliance, including trust in science [23], fear of COVID-19 [24], and conspiracy thinking [25]. Another factor explored has been having a left versus a right-wing political orientation, which is the primary focus of the present study.

When people think about the similarities and differences between political orientations, they are often drawn to a spatial metaphor: the left-right “spectrum.” This spectrum, often used interchangeably with a liberal-conservative one, especially in U.S. context, is an organizing device that helps to define how different ideologies relate to each other. Those on the left tend to support expanded government and increased spending, whereas those on the right usually support smaller government and reduced spending [26]. This spectrum has been used in relevant research on morality and politics in both North American [27] and European contexts [28], providing a meaningful way of pooling and comparing data from different nations. In turn, there is evidence of similar links between the left-right political spectrum and COVID-19 related attitudes and behaviours in U.S. and Canadian contexts.

Political polarization along the left-right spectrum during the pandemic has been evidenced in numerous domains, including the advice given around adherence with public health guidelines by political leaders and media outlets [29], and in terms of individual compliance. Studies conducted during the first wave of lockdowns in the U.S. examining COVID-19 health protective behavioural intentions found that left-wingers were more likely than right-wingers to report willingness to comply with government-imposed COVID-19 restrictions and health protective behaviours [10]. Conversely, right-wing oriented individuals reported more defiant intentions in relation to social distancing [30]. Similar findings have been reported in the Canadian context, with political preferences across the spectrum similarly related to a COVID-19 compliance measure including handwashing, mask-wearing, avoidance of public spaces, and social distancing [31].

Another domain of political difference around social distancing measures has been whether governments responses were seen as over-reactions or under-reactions. For example, in a comparative analysis of COVID-19 attitudes and behaviours in the U.S. and Canada, it was found that Democrats were significantly more likely than Republicans to say it was a slight or significant underreaction [32]. There were also statistically significant differences between Liberals and supporters of all other parties in Canada, again closely aligned with the left-right spectrum. These differences in turn relate to wider debates about the relative prioritization of health versus economic concerns, with those on the left placing more relative weight on the former than those on the right [33]. What factors might explain these political differences? One possibility is that they relate to people’s underlying moral values.

Moral Foundations Theory [34–37] provides a promising means for exploring relationships between attitudes and moral value systems. MFT is based on the notion that intuitive moral values influence moral judgments and behaviours [38] often without conscious thought [35]. One or more moral foundations can be activated in any given situation [39], in which case the relative endorsement of each one could affect judgment and behaviour [40].

MFT was originally comprised of five moral foundations, and a sixth foundation has been proposed. The first, care/harm, underlies virtues of kindness, gentleness, nurturance, and harm prevention. The second, fairness/cheating, generates ideas of justice, rights, and reciprocity. The third, loyalty/betrayal, underpins virtues of patriotism and self-sacrifice for the ingroup. The fourth, authority/subversion, includes deference to legitimate authority, and respect for traditions and hierarchies. The fifth is purity/degradation and emphasizes bodily and moral purity. A sixth proposed foundation is liberty/oppression, which relates to feelings of reactance and resentment toward those who restrict freedoms. This in turn has been subdivided into economic and lifestyle liberty, the former stressing the importance of freedoms such as people’s individual property rights, the latter including the freedom for people to choose what they do [41].

Of central relevance to the present study, the relative endorsements of these moral values differ between those on the political left and right. A review of the literature, conducted in multiple countries, indicates that those on the left of the political spectrum more heavily endorse the values of care and fairness than the other foundations [34]. Care and fairness have been termed the “individualizing foundations” as they emphasize individual rights, wellbeing, and protection [34, 42]. In contrast, those on the political right tend to value the foundations of purity, loyalty, authority as much as those of care and freedom. These “binding foundations” primarily view individuals as duty-bound, socially connected members within their groups or institutions [43]. There are also differences on the liberty/oppression foundation, with those on the right more strongly endorsing the economic liberty items, and a lack of difference on lifestyle liberty [41].

So, there are consistent moral values differences across the left-right political spectrum, and these may be relevant to the relationship between political orientation and social distancing. Firstly, social distancing restrictions elicit a potential clash between the individualizing and binding foundations. If everyone is ordered to comply, someone on the left who values the individualizing foundations over the binding ones will likely do so, in fairness to others and to minimize the harms of viral transmission [44]. Although those on the political right do still value care, they differ regarding to what degree it needs to be balanced with other moral foundations [40]. As they place more equal value on the importance of care for others versus maintaining social connections within groups and institutions [43], then the arguments in favour of social distancing from their perspective may be less reasonable [45].

Compliance with social distancing regulations also imposes significant restrictions on individual freedoms as it requires people to limit their range of typical interactions. Thus, the liberty/oppression foundation is also germane here [46]. As those on the political right value liberty, especially economic liberty, more strongly than those on the left [41], then they may view the negative economic effects of social distancing measures like lockdowns as too injurious, and perhaps as outweighing their health benefits [33]. In contrast, the moral calculus of those on the left will more often see the need to protect people from harm as outweighing the need to preserve economic freedoms, for example by opening up the economy sooner. Consistent with this, people on the political left are more likely to moralize compliance with COVID-19 social distancing specifically [47], and public health orders in general [48].

So, to what degree do the findings support these theoretical assertions? Several studies conducted in the early months of the pandemic show links between people’s endorsement of moral foundations and their past or intended future compliance with composite self-report measures, including items related to hygiene-related and social distancing behaviours. Consistent links between self-reported compliance and the care/harm foundation have been found in samples in the U.K, [24], in the U.S., and France [46], and in Japan [49]. Moreover, there is some evidence of a negative association between valuation of liberty items and compliance [44, 46]. The evidence regarding the other foundations has been more mixed, albeit with a positive link reported between compliance and fairness [46], and a negative association with endorsement of the authority foundation [49].

Of course, a reason for this mixed evidence could be the differing national contexts in which the research has taken place, and so the present study seeks to explore the trends across two national contexts to determine their consistency. It may be because the composite measures used included distinct preventive behaviours, which may be relevant to different moral foundations [10]. Given the focus of the current study, it is thereby prudent to explore past findings isolating the relationships between the moral foundations and social distancing behaviours in particular.

Self-reported willingness to comply with stay-at-home, social distancing, and mask-wearing guidelines were tested in a study in April 2020 in the U.S. [10]. Greater endorsement of the individualizing moral foundations of care and fairness was positively related to all three preventive behaviours, whereas endorsement of the purity moral foundation was negatively related to both social distancing and mask-wearing compliance [10]. Similarly, in another U.S. sample surveyed late in March 2020 reported that endorsement of the individualizing foundations was negatively predictive of intentions to defy social distancing guidelines [30]. Conversely, valuation of the binding foundations was positively associated with intended defiance.

So, the few studies that have isolated compliance with social distancing guidelines suggest that the valuation of the individualizing foundations is linked to more compliance, and that the converse is the case for the binding foundations. There is a relative lack of information around the liberty foundation, although some studies are suggestive of such a link [44, 46]. As these relationships are like those found between political orientation and both moral foundations and social distancing compliance, it is possible that these behavioural differences across the political spectrum are partly accounted for by differing endorsement of moral foundations.

This proposition, yet to be tested directly, will be investigated in the present study. In comparison to past research sampling short-term compliance, it will use a broad range of social distancing outcome measures, including those addressing attitudes, longer-term behavioural compliance, and wider perceptions of relevant government responses and priorities during the pandemic. Moreover, the later-pandemic timing of the present study offers a window into these relationships as the countries surveyed are pursuing mass vaccination programs, and gradual movement away from social distancing requirements [50].

The main hypotheses, based on past findings and a consideration of central tenets of MFT, are as follows:

H1: A more left-wing political orientation will positively correlate with endorsement of the individualizing moral foundations, negatively correlate with endorsement of the binding and economic liberty foundations, and be uncorrelated with endorsement of the lifestyle liberty items.
H2: A more left-wing political orientation will positively correlate with participants’ social distancing attitudes, self-reported compliance behaviours, moralization of social distancing compliance and of public health, lower perceptions of government overreaction, and greater endorsement of health versus economic prioritization.
H3. Participants’ endorsement of individualizing moral values (harm/care and fairness) will positively correlate with their social distancing attitudes, self-reported compliance behaviours, moralization of social distancing compliance and of public health, lower perceptions of government overreaction, and greater endorsement of health versus economic prioritization.
H4. Participants’ endorsement of binding moral values (authority, purity, and loyalty) and economic liberty items will negatively correlate their social distancing attitudes, self-reported compliance behaviours, moralization of social distancing compliance and of public health, lower perceptions of government overreaction, and greater endorsement of health versus economic prioritization.
H5. The relationship between participants’ political orientation and social distancing attitudes, self-reported compliance behaviours, moralization of social distancing compliance and of public health, lower perceptions of government overreaction, and greater endorsement of health versus economic prioritization will be significantly mediated by differences in their endorsement of individualizing, binding, and economic liberty moral foundations.

It is possible that the outcomes of this exploratory study will contribute to the understanding of preventive behaviours, not only during the ongoing COVID-19 crisis, but during other public health crises that may arise in the future. By examining moral foundations associated with social distancing compliance, public health authorities can communicate the importance of such actions based on individuals’ favoured moral foundations, in the hope of increasing behavioral compliance [10, 51].

To that end, the present study will also explore whether there is a relationship between participants’ political orientation, moral foundations, and how persuasive they find messages supporting ongoing social distancing compliance framed on each of the six moral foundations. These messages will be based on an important narrative at the time of writing, namely that some ongoing distancing measures are likely to be needed, until vaccination rates are sufficient to reach a level of herd immunity. This builds on previous research showing that people are more persuaded by arguments reframed in terms of the moral values they most endorse [52]. This is, to our knowledge, the first study to explore moral reframing in the context of social distancing arguments, and so there are no formal hypotheses here. As vaccination uptake and hesitancy are relevant to this narrative, the present study will also include a measure COVID-19 vaccine hesitancy. Again, no specific hypotheses will be made here, given the preliminary nature of this part of the study. The results of these exploratory analyses are reported in the Supplementary Materials section.

Ultimately, it is the intent of this study to explore whether MFT offers a pathway for clarifying the interwoven relationships between political orientation, moral values, and social distancing.

## Materials and methods

### Participants

After securing ethical approval from the Research Ethics Board at Kwantlen Polytechnic University, participants were recruited through a psychology department research participant pool, offering bonus course marks, at a large, Western Canadian university, and through the online platforms of the Social Psychology Network, and Reddit SampleSize. The study was conducted utilizing the Qualtrics survey platform and took respondents about 20 minutes to complete. The survey was live from April to June 2021, at a time when the spread of the Delta variant was just starting in Canada and the U.S., and when people had been living with various social distancing measures for over a year. A total of 303 individuals completed at least a portion of the study. Of these, 209 provided complete data sets for all key predictor and outcome variables, a further 5 completed the questionnaires but then requested their data be withdrawn, 6 respondents failed at least one of the two attention check questions, with both these latter groups’ data excluded from the analyses. 83 participants provided partial data, and a further 11 participants were ineligible based on either the initial age or residency questions. A series of independent samples *t* tests were run, comparing the mean scores of the complete versus partial data groups, and revealed no significant differences on any of the key demographic variables of age, political orientation, most recent individual and household incomes, highest level of education attained, and whether they had ever received a positive test for COVID-19. On this basis, it was concluded that the incomplete data sets were not significantly different than the complete ones, and that the attrition had likely not biased the sample demographics. The incomplete data sets were thereby excluded from further analyses. From the complete data set, (N = 209) the mean age was 28.56 years (SD = 10.62), with a range from 18 to 71 years old. 109 (52%) reported residence in the U.S., and 100 (48%) in Canada. To maximize inclusivity, participants indicated their gender and ethnicity in open-ended questions [53] and then grouped using U.S. and Canadian census categories wherever possible (see Table 1 for the distributions). For statistical purposes, based on empirical and theoretical considerations, gender was then recoded as male vs. nonmale, and ethnicity as white vs. nonwhite [10].

**Table 1:** Descriptive statistics for demographic and descriptive political measures.

Characteristics		
Gender (N = 207)	Female	126 (%)
	Male	67 (%)
	Genderfluid/Agender	8 (%)
	Non-binary/Agender	5 (%)
	Transgender	2 (1%)
Ethnicity (N = 205)	White	141 (%)
	South Asian	24 (%)
	Asian	23 (%)
	Mixed	8 (%)
	Hispanic/Latino/Spanish origin	6 (%)
	Black/African American	2 (%)
	Indigenous/First Nations/American Indian/Alaska Native	2 (%)
Education (N = 209)	Less than high school	2 (1%)
	High school graduate	68 (32.5%)
	Two-year associate or associate degree	36 (17.2%)
	Four-year college degree	63 (30.1%)
	Master’s or professional degree	25 (12%)
	Doctoral degree	15 (7.2%)
Income (US$) (N = 176)	Individual	M = $38 640, SD = $38 779
	Household	M = 100 968, SD = 88, 783
Ever received positive COVID-19 test (N = 209)	Yes	6 (12%)
	No	203 (88%)
Political party identification–U.S. sample (N = 109)	Democrat	62 (57%)
	Republican	11 (10%)
	Independent	23 (21%)
	Other	13 (12%)
Voted in most recent federal election–U.S. sample (N = = 109)	Yes	98 (90%)
	No	11 (10%)
Party voted for in most recent federal election–U.S. sample (N = 98)	Democrat	74 (76%)
	Republican	14 (14%)
	Independent	4 (4%)
	Other	6 (6%)
Political party identification–Canadian sample (N = 100)	New Democratic Party of Canada	40 (40%)
	Liberal Party of Canada	31 (31%)
	Conservative Party of Canada	8 (8%)
	Bloc Quebecois	1 (1%)
	Green Party of Canada	13 (13%)
	Other	7 (7%)
Voted in most recent federal election–Canadian sample (N = 83)	Yes	74 (89%)
	No	9 (11%)
Party voted for in most recent federal election–Canadian sample (N = 83)	New Democrat Party of Canada	27 (37%)
	Liberal Party of Canada	31 (42%)
	Conservative Party of Canada	5 (7%)
	Bloc Quebecois	1 (1%)
	Green Party of Canada	8 (11%)
	Other	2 (3%)

### Measures and procedure

#### Political orientation

After completing the demographics questions, participants were asked to indicate their political views on a 7-point scale ranging from “1 –Very left-wing” to “4 –Moderate” to “7 –Very right-wing”. This type of scale has been widely used in related research [27, 28, 43].

They were then asked, based on their country of residence, to indicate which one of the federal political parties they most identified with, whether they had been eligible to vote in the most recent federal elections, and if so, which party they had voted for. The response options for the Canadian resident sample were as follows: “The Conservative Party of Canada”; “The Bloc Québécois”; “The Liberal Party of Canada”; “The New Democratic Party (NDP)”; “The Green Party of Canada”; “Other (please specify)”. The options for the U.S. resident sample were: “Republican”; “Democrat”; “Independent”; “Other (please specify)”.

Interestingly, although 57% reported, in the United States, to be Democrats, the 1–7 point scale on political views, shows a considerable range of responses variety, albeit still predominantly on the left. 69.7% stated they were either very-left wing (28.4%), moderately left-wing (32.1%), or slightly left-wing (9.2%), Still, 14.7% say they were moderate, and more than 15% said their political views were either slightly right-wing (8.3%), moderately right-wing (4.6%) or very right-wing (scale 7 on the 1–7 point scale, at 2.8%). The Canadian sample also consisted mainly of respondents who identified with parties of the left and center-left, namely the NDP (40%) or the Liberal Party (31%). The 1–7 political orientation scale indicated that 68% responded as either very left, moderately left or slightly left. 22% said they were moderate (point 4 on the scale), while 10% indicated a score on the right. As such, the simple party identification items did not give the full picture of the variation in political views in the overall sample.

#### Moral foundations questionnaire

The MFQ30 (the 30 items Moral Foundations Questionnaire) was used in the present study. It consists of 30 statements covering five moral foundations (care/harm, fairness/reciprocity, authority/ respect, ingroup/loyalty, purity/sanctity; six items per foundation) using two different response formats. The first section asked participants to rate the relevance of a particular domain when they make a moral decision (“Whether or not someone acted unfairly”; fairness foundation). The response scale ranged from “0—Not at all relevant” to “5—Extremely relevant,” The second section asked participants to rate their endorsement of a range of moral propositions (“I am proud of my country’s history”; loyalty foundation). Here, the response scale was as follows: “0—Strongly disagree” to “5—Strongly agree.” This was scored functionally from 0 to 5.

Following previous work expanding the MFQ to include liberty, nine items for measuring endorsement of the liberty foundation were also included, within the respective sections of the MFQ [41, 54]. Also in line with this previous work, six items were used to form the economic/government liberty subscale (e.g., ‘‘People who are successful in business have a right to enjoy their wealth as they see fit”). The remaining three comprised the ‘‘lifestyle liberty” subscale (e.g., ‘‘Everyone should be free to do as they choose, as long as they don’t infringe upon the equal freedom of others”).

Two additional items were fillers, not used in calculating the foundation scores: “Whether or not someone was good at math” and “It is better to do good than to do bad”. These were used as attention checks to exclude inattentive participants.

Responses were averaged for each moral foundation, with higher scores indicative of greater endorsement. Foundation scores showed acceptable internal consistency, comparable to those from previous research: Care (α = .70), Fairness (α = .68), Loyalty (α = .75), Authority (α = .81), Purity (α = .86), Government/Economic Liberty (α = .79), Lifestyle liberty (α = .68).

Following previous research [30, 55, 56] the three group-focused indices (authority, purity, and loyalty) were averaged to generate an overall binding foundations index (α = .92), and the average of the two individual-focused indices (care and fairness) was used to generate an overall individualizing foundations index (α = .81).

#### Social distance attitudes scale

The Social Distance Attitudes Scale (SDAS) is a 14-item questionnaire measuring attitudes expressing either support for (8 items) or opposition to (6 items) social distancing, which can form separate “positive” and “negative” subscales [57]. The supportive items include those focusing on importance of social distancing to reduce transmission of the virus, to preserve healthcare capacity, and the need to continue it until additional public health infrastructure becomes available. The opposition items included ones focused on social distancing policies violating individual rights and social distancing not being beneficial. Participants indicated their attitudes ranging from “1 –Strongly disagree” to “5 –Strongly agree”. The SDAS subscales have high internal consistency, construct, and predictive validity [57]. In the current sample, though, the two subscales were highly intercorrelated (*r*(207) = -.90, p < .001), and so the opposition items were reverse scored, and a single scale was then computed, with higher scores indicating more positive overall attitudes towards social distancing. This single scale showed high internal consistency in the current sample (α = .95).

#### Social distancing compliance scale

A scale measuring compliance with social distancing rules since their inception was developed, as extant scales were more short-term (often sampling a week or so). A range of social distancing rules were included based on relevant literature and guidelines. Participants were asked about their following of these rules since the beginning of the COVID-19 pandemic, when they had been in place where they lived (this was to allow for geographical/jurisdictional variations in the timing of imposition and relaxing of the various rules). To ensure their relevance to all participants, the items selected covered restrictions that had been imposed throughout North American jurisdictions. Example items include: “Avoiding non-essential gatherings (e.g., social events)”, “Keeping the recommended safe distance from people who do not typically live with you”, and “Avoiding non-essential travel (domestic, international)”. For each item, participants responded to the question “How much have you followed this rule, when it has been in place where you live?” The following response scale was used: “1 = Not at all” to “8 = Always” [58]. In a factor analysis, using principal axis factoring and direct oblimin rotation, scores loaded onto a single factor (loadings ranging from .67 to .91). On this basis, scores were averaged across the 9 items, creating an overall past compliance score, with higher scores indicating greater compliance (α = .94).

An additional item asked participants to indicate their intended future compliance, phrased in the following way: “From now on, how much do you intend to follow social distancing rules in general?” The same 8-point response scale was used as with the past compliance items. Finally, participants were asked: “How much longer would you be willing to follow some form of social distancing rules from now?”, using the following response scale: “< 1 month / 1–3 months / 4–6 months / 7–9 months / 10–12 months / > 12 months”. These responses were then recoded onto a scale from 1–6.

#### Moralization scale

Next, two questions regarding the extent to which participants moralized social distancing compliance and public health were included. The first was “To what extent do you feel that violating social distancing rules is morally condemnable (i.e., how "wrong" is such behavior)? Responses ranged on a scale from “1 = totally acceptable to violate social distancing rules” to “5 = totally unacceptable to violate social distancing rules” [48]. Next, they were asked. “To what extent do you think that public health is a moral issue?”, using a response scale ranging from “1 = Not at all” to “5 = Extremely” [47]. Due to their being significantly intercorrelated in the present sample (*r*(207) = .48, p < .001), and because of their conceptual overlap, the two items were combined to create a moralization scale, albeit one with modest internal consistency (α = .65).

#### Government overreaction to COVID-19 scale

A scale was developed based on an existing measure [58] assessing whether participants viewed their government’s impositions of various widespread social distancing restrictions as underreactions versus overreactions to the pandemic. Sample items include: “Restrictions on social contacts outside of household members”; “Travel restrictions (domestic and international)”; “Closing of cultural and sporting events and facilities”. The following response scale was used: “1 = Significant underreaction”, “2 = Slight underreaction”, “3 = Appropriate reaction”, “4 = Slight overreaction”, “5 = Significant overreaction”.

In a factor analysis, using principal axis factoring and direct oblimin rotation, scores loaded onto a single factor (loadings ranging from .49. to .83). The scores were thus averaged across the 8 items, creating an overall government overreaction score, with higher scores indicating a greater perception of overreaction (α = .89).

#### Attitudes towards health vs economic prioritization scale

Participants were asked to indicate their level of agreement/disagreement with four items, based on the content of wider, politicized debates about balancing health versus economic priorities during COVID-19 [59]. The following response scale was used: “1 –Very strongly disagree”, “2 –Strongly disagree”, “3 –Disagree”, “4 –Neither agree nor disagree”, “5 –Agree”, “6 –Strongly agree”, “7 –Very strongly agree”). Two items were framed in terms of prioritizing health concerns (e.g., “At this stage of the pandemic, the highest government priority should be to save as many lives as possible, despite this slowing the economic recovery”), and the other two were framed as prioritizing economic concerns, (e.g., “At this stage of the pandemic, I am more concerned about the economic impact of the COVD-19 outbreak than the public health impact”). The two prioritizing economic concerns were reverse scored, and the four items were factor analyzed using principal axis factoring and direct oblimin rotation. The scores loaded onto a single factor (loadings ranging from .86 to .91). and a composite score was calculated measuring relative prioritization of health versus economic concerns (α = .93).

#### Vaccine hesitancy

Participants were asked two initial questions, which were coded dichotomously. The first was: “Have you had the opportunity to receive a COVID-19 vaccination yet?”, with a “Yes” or “No” response format. 133 (64%) responded “yes” to this question. The second was only displayed if participants answered yes to the first question: “If yes, did you choose to get the vaccination or to refuse it?” with the response options of “Yes, got the vaccination” and “No, refused the vaccination”. Of those 133 participants, 126 (95%) responded “yes”, and 7 (5%) responded “no”.

Only the 76 participants who answered “No” to the first question were presented with Oxford Covid-19 Vaccine Hesitancy Scale, as this is aimed at sampling attitudes of those who have yet to receive a vaccination [60]. This is a seven-item measure using item specific response options, coded from 1 to 5, are used. A ‘Don’t know’ option is also provided, which is excluded from scoring. Sample items include “Would you take an COVID-19 vaccine if offered?” (with the following response options: “1 = Definitely”, “2 = probably”, “3 = I may or may not”, “4 = Probably not”, “5 = Definitely not”, and “Don’t know”), and “I would describe my attitude towards receiving a COVID-19 vaccination as:” (with the following response options: “1 = Very keen”, “2 = Pretty positive”, “3 = Fairly neutral”, “4 = Quite uneasy”, “5 = Against it”, and “Don’t know”). Higher scores indicate a higher level of vaccine hesitancy. The scale shows a unifactorial structure, with high internal consistency and strong convergent validity with existing measures of vaccine hesitancy [60]. The internal consistency was high in the present sample (α = .97).

#### Moral arguments

Finally, participants read seven short paragraphs containing moral arguments about why following social distance rules until most people have been vaccinated and herd immunity is reached is important, a key narrative supported by many medical experts at the time [61]. These were presented at the end of the questionnaire, so that they did not contaminate participants’ responses to earlier questions. The argument paragraphs were each based on one of the moral foundations, namely care, fairness, loyalty, authority, purity, and liberty, using key words from the moral foundations dictionary [62]. The length of each paragraph and the number of key words used from each foundation were held similar. A seventh, morally neutral paragraph of equal length, using only factual information about COVID-19 was also included, based on one rated as highly persuasive in a previous study [63]. The following are example arguments for following social distancing rules from each paragraph. Care: “We are showing compassion for those who are most vulnerable.” Fairness: “We are being fair to everyone, as we all have an equal right to be protected from the virus as much as possible.” Loyalty: “We all need to do this for our families, friends, and fellow citizens.” Authority: “It is critical to continue to defer to the authority of our leaders during this crisis.” Purity: “We need to continue to follow the rules to keep our bodies as pure and our communities as clean as possible from this repulsive virus!” Liberty: “These guidelines are the best way to return individuals to liberty, with the freedom to do what they want.” Control: “We can help prevent the spread of COVID-19 by following social distancing guidelines.”

Following each paragraph, participants were asked how “persuasive” and “convincing” the message was, and how much it “made its point” [47, 64]. The order that participants saw each paragraph was fully randomized, and the instructions asked them to consider each paragraph separately. All responses were a on 5-point scale (1 = Not at all, 5 = extremely). As the items were highly intercorrelated, they were averaged to form a composite persuasiveness measure for each moral foundation paragraph (harm α = .91, fairness α = .91, loyalty α = .91, authority α = .86, purity α = .89, liberty α = .91, control α = .90).

### Analytic plan

The analysis comprised four phases. First, key descriptive statistics were calculated regarding the outcome variables (e.g., social distancing attitudes) and predictor variables (i.e., political orientation, moral foundations). Second, to test hypotheses 1–4, Pearson product moment correlations were calculated between them. Then, to test hypothesis 5, multiple mediation analyses were conducted on the following outcome variables: (a) social distancing attitudes (b) social distancing past compliance (c) social distancing future compliance, d) moralization of social distancing compliance and public health, (e) attitudes towards government reactions, (f) attitudes towards health vs. economic prioritization, and (g) vaccine hesitancy. Such mediation analyses are consistent with those undertaken in similar work assessing the mediating influence of the moral foundations on the relationship political orientation and attitudes to other socially important issues, including human rights attitudes [43].

Finally, exploratory analyses were performed to assess any relationships between political orientation, moral values, and the perceived persuasiveness of arguments in favour of ongoing social distancing.

An *a priori* power analysis was performed using G*Power 3.1.9.7 [65], to determine the required sample size for a medium (*f*_2_ = .15) effect size for a linear multiple regression. This was done with 4 predictors and an alpha level of *p* < .05. This indicated that minimum sample size of 129 participants was required. A further analysis was performed to determine the required sample size for a medium (*r* = .30) effect size for bivariate correlations, with an alpha level of 0.05. This indicated that minimum sample size of 115 participants was required. In both cases, the obtained overall sample size of 209 was thus adequate for these purposes.

## Results

### Data preparation

All data analysis was conducted using SPSS Statistics, Version 24. Tables 1 and 2 show the descriptive statistics for the demographic, and predictor and outcome variables, respectively.

**Table 2:** Descriptive statistics for key predictor and outcome measures (N = 209).

Measure	M	SD	Range	
			Possible	Actual
Political orientation	2.74	1.50	1–7	1–7
Individualizing foundations	3.76	0.67	0–5	1.58–5
Binding foundations	2.02	0.93	0–5	0.22–5
Government/economic liberty	2.63	1.02	0–5	0.33–5
Lifestyle liberty	3.56	0.87	0–5	1–5
SDAS	4.20	0.87	1–5	1–5
SDCS–P	6.60	1.42	1–8	1–8
SDCS–FIC	6.20	1.94	1–8	1–8
SDCS–FDC	4.25	1.72	1–6	1–6
MS	3.66	1.12	1–5	1–5
GOS	3.09	0.65	1–5	1–5
HVEPS	5.03	1.52	1–7	1–7
OCVHS (N = 66)	2.11	0.84	1–5	1.57–4.86

Key: SDAS: Social Distancing Attitudes Scale; SDC-P; Social Distancing Compliance Scale–Past; SDCS-FIC: Social Distancing Compliance Scale–Future Intended Compliance; SDC-FDC: Social Distancing Compliance Scale–Future Duration of Compliance; MS: Moralization Scale; GOS–Government Overreaction Scale; HVEPS: Health versus Economic Prioritization Scale. OCVHS–Oxford Covid Vaccine Hesitancy Scale.

### Correlational analyses

To test hypotheses 1–4, Pearson product-moment correlations were calculated among the key political, moral, and social distancing outcomes. The significance of the observed correlations was then determined, as shown in Tables 3–8.

**Table 3:** Correlation matrix for key political and moral variables (n = 209).

Variable	PO	IMF	BMF	ELMF	LLMF
Political Orientation	-	-.43**	.62**	.62**	-.02
Individualizing Moral Foundations	-	-	-.10	-.30**	-.23*
Binding Moral Foundations	-	-	-	.54**	-.02
Economic Liberty Moral Foundation	-	-	-	-	.33**
Lifestyle Liberty Moral Foundation	-	-	-	-	-

Key: PO: Political Orientation; IMF: Individualizing Moral Foundations; BMF: Binding Moral Foundations; ELMF: Economic Liberty Moral Foundation; LLMF: Lifestyle Liberty Moral Foundation

* *p* < .01 (two-tailed).

** *p* < .001 (two-tailed).

**Table 4:** Correlation matrix for key political and moral variables in Canadian sample (n = 100).

Variable	PO	IMF	BMF	ELMF	LLMF
Political Orientation	-	-.30**	.55***	.55***	.15
Individualizing Moral Foundations	-	-	.06	-.06	.29**
Binding Moral Foundations	-	-	-	.54***	.22*
Economic Liberty Moral Foundation	-	-	-	-	.56***
Lifestyle Liberty Moral Foundation	-	-	-	-	-

Key: PO: Political Orientation; IMF: Individualizing Moral Foundations; BMF: Binding Moral Foundations; ELMF: Economic Liberty Moral Foundation; LLMF: Lifestyle Liberty Moral Foundation

* *p* < .05 (two-tailed).

** *p* < .01 (two-tailed).

*** *p* < .001 (two-tailed).

**Table 5:** Correlation matrix for key political and moral variables in U.S. sample (n = 109).

Variable	PO	IMF	BMF	ELMF	LLMF
Political Orientation	-	-.55***	.69***	.69***	-.15
Individualizing Moral Foundations	-	-	-.25**	-.48***	.19
Binding Moral Foundations	-	-	-	.54***	-.26**
Economic Liberty Moral Foundation	-	-	-	-	.13
Lifestyle Liberty Moral Foundation	-	-	-	-	-

Key: PO: Political Orientation; IMF: Individualizing Moral Foundations; BMF: Binding Moral Foundations; ELMF: Economic Liberty Moral Foundation; LLMF: Lifestyle Liberty Moral Foundation

* *p* < .05 (two-tailed).

** *p* < .01 (two-tailed).

*** *p* < .001 (two-tailed).

**Table 6:** Correlation matrix for political, moral, and social distancing outcome measures (n = 209).

	SDAS	SDCS-P	SDCS-FIC	SDCS-FDC	MS	GOS	HVEPS
Political Orientation	-.59**	-.46**	-.40**	-.34**	-.54***	.52**	-.63**
Individualizing Moral Foundations	.40 **	.28**	.32**	.22*	.33***	-.30**	.43**
Binding Moral Foundations	-.42 **	-.37**	-.24**	-.24*	-.36***	.33**	-.53**
Economic Liberty Moral Foundation	-.53 *	-.41**	-.35**	-.33**	-.46***	.47**	-.64**
Lifestyle Liberty Moral Foundation	-.12	-.07	.09	-.04	-.08	.10	-.05

Key: SDAS-P: SDAS: Social Distancing Attitude Scale; SDCS-P; Social distancing compliance–past; SDCS-FIC: Social Distancing Compliance–Future Intended Compliance; SDCS-FDC: Social Distancing Compliance–Future Duration of Compliance; MS: Moralization Scale; GOS–Government Overreaction Scale; HVEP: Health versus Economic Prioritization Scale.

* *p* < .01 (two-tailed).

** p < .001 (two-tailed).

**Table 7:** Correlation matrix for political, moral, and social distancing outcome measures in Canadian sample (n = 100).

	SDAS	SDCS-P	SDCS-FIC	SDCS-FDC	MS	GOS	HVEPS
Political Orientation	-.52***	-.38***	-.36***	-.26**	-.46***	.53***	-.58***
Individualizing Moral Foundations	.14	.03	.32**	.13	.16	-.15	.23*
Binding Moral Foundations	-.43***	-.29**	-.23*	-.20*	-.24*	.33**	-.54***
Economic Liberty Moral Foundation	-.50***	-.38***	-.31**	-.37***	-.43***	.54***	-.61***
Lifestyle Liberty Moral Foundation	-.26**	-.23*	-.14	-.14	-.14	.27**	-.20

Key: SDAS-P: SDAS: Social Distancing Attitude Scale; SDCS-P: Social Distancing Compliance Scale–Past; SDCS-FIC: Social Distancing Compliance Scale–Future Intended Compliance; SDCS-FDC: Social Distancing Compliance–Future Duration of Compliance; MS: Moralization Scale; GOS–Government Overreaction Scale; HVEPS: Health versus Economic Prioritization Scale.

* *p* < .05 (two-tailed).

** *p* < .01 (two-tailed).

*** *p* < .001 (two-tailed).

**Table 8:** Correlation matrix for political, moral, and social distancing outcome measures in American sample (n = 109).

	SDAS	SDCS-P	SDCS-FIC	SDCS-FDC	MS	GOS	HVEPS
Political Orientation	-.59***	-.51***	-.43***	-.38***	-.61***	.51***	-.68***
Individualizing Moral Foundations	.59***	.42***	.44***	.38***	.49***	-.40***	.56***
Binding Moral Foundations	-.44***	-.47***	-.31**	-.29**	-.25**	.34***	-.54***
Economic Liberty Moral Foundation	-.55***	-.44***	-.39***	-.30**	-.48***	.43***	-.66***
Lifestyle Liberty Moral Foundation	-.01	.04	-.06	.06	.19	-.02	.05

Key: SDAS-P: SDAS: Social Distancing Attitude Scale; SDCS-P: Social Distancing Compliance Scale–Past; SDCS-FIC: Social Distancing Compliance Scale–Future Intended Compliance; SDCS-FDC: Social Distancing Compliance Scale–Future Duration of Compliance; MS: Moralization Scale; GOS–Government Overreaction Scale; HVEPS: Health versus Economic Prioritization Scale.

* *p* < .01 (two-tailed).

** p < .001 (two-tailed).

*** *p* < .001 (two-tailed).

As can be seen in Table 3, in line with hypothesis 1, and consistent with previous research, with a left-wing orientation positively correlated with degree of endorsement of individualizing moral foundations, negatively correlated with the binding foundations and government/economic liberty items, and unrelated to endorsement of the lifestyle liberty items [41]. These correlational analyses were then computed within both the Canadian and U.S. samples, to check for consistency of findings across these two different national contexts. The results are shown in Tables 4 and 5. As can be seen, overall, the pattern of significant correlations between political orientation and the moral foundations was consistent in both samples, and supportive of hypothesis 1.

As depicted in Table 6, there were significant correlations in the moderate range between political orientation, and each of the social distancing outcome measures, in support of hypotheses 2–4.

Overall, a more left-wing political orientation was significantly associated with more positive and less negative social distancing attitudes, greater past compliance, greater future intended compliance, more moralization of compliance and public health, lower perceptions of government overreaction, and a greater prioritization of health over economic concerns. As predicted, this pattern of correlations each of the key outcome measures and valuation of both the binding and economic liberty foundations. Also as hypothesized, the opposite pattern of correlations was observed regarding valuation of the individualizing foundations. Further, as anticipated, endorsement of the lifestyle liberty items was not significantly correlated with any of the key outcome measures. These results also obtained largely across the two national samples, as depicted in Tables 7 and 8. Overall, the pattern of significant correlations was consistent and in line with hypotheses 3 and 4. The exception to this was that individualizing foundations scores were significantly correlated with all of the social distancing outcome measures in the U.S. sample, but, counter to hypothesis 3, only with the HVEP scale in the Canadian sample.

Preliminary analyses showed that vaccine hesitancy across the whole sample, as measured by the Oxford Covid-19 Vaccine Hesitancy scale, was negatively correlated with political orientation (*r*(65) = .41, p < .001), with those more on the right more hesitant, and positively associated with endorsement of the binding (*r*(65) = .39, p < .001), and economic liberty moral foundations (*r*(65) = .43, p < .001). Vaccine hesitancy was not significantly related to the individualizing foundations (*r*(65) = -.14, p = .28).

### Mediation analyses

To test hypothesis 5, namely whether the relationships between political orientation and the social distancing outcome measures were mediated by moral foundations scores, a series of multiple mediation regression analyses were run regarding each key outcome variable separately, as they were all addressing different aspects of social distancing responses. For each model, tests for violations of assumptions, bias, and multicollinearity were conducted [66]. Based on examination of relevant diagnostic statistics, no multicollinearity, outliers, residuals, or influential cases were found. As a result, the analyses were conducted using the complete dataset (N = 209). The analyses were run with the Process SPSS Macro version 2.15 [67] with 5,000 bootstrap samples. For each analysis, the initial intention was to run a hierarchical regression with the demographic variables in step 1, but since they were found to be non-significantly correlated with any of the outcome variables, they were not included. Political orientation was entered as the independent variable, the individualizing, binding, and economic liberty moral foundations scores as the mediator variables, and the specific social distancing outcome measure used each time as the dependent variable. Results are reported here following relevant guidelines [43, 66, 67].

In each mediation model, political orientation had a significant negative effect on the individualizing moral foundations (*b* = -.19, *t*(207) = −6.78, p < .0001, *R*^2^ = .18), a significant positive effect on the binding moral foundations (*b* = .39, *t*(207) = 11.45, p < .0001, *R*^2^ = .39), and a significant positive effect on the economic liberty moral foundation (*b* = .42, *t*(207) = 11.21, p < .0001, *R*^2^ = .38).

Separate mediation analyses were run for each outcome variable, with the results for each shown in Tables 9–15.

**Table 9:** Mediation effects of moral foundations on the relationship between political orientation and social distancing attitudes scale–past (N = 209).

Predictor	*B*	SE	*b* 95% CI [LL, UL]	*t*	*p*	*R* ^*2*^
Constant	4.48	.36	[3.77, 5.19]	12.40	.000	
PO Total Effect	-.34	.03	[-.41, -.28]	-10.41	.000	
Direct Effects						
PO	-.18	.05	[-.28, -.09]	-3.79	.000	
IMF	.24	.08	[.08, .40]	3.03	.003	
BMF	-.08	.07	[-.21, .06]	-1.09	.275	
ELMF	-.20	.06	[-.32, -.08]	-3.34	.001	
						.41
Indirect Effects						
Total	-.16	.03	[-.22, -.09]			
**IMF**	**-.05**	**.02**	**[-.08, -.01]**			
BMF	-.03	.02	[-.07, .02]			
**ELMF**	**-.09**	**.03**	**[-.14, -.03]**			

Key: PO: Political Orientation; IMF: Individualizing Moral Foundations; BMF: Binding Moral Foundations; ELMF: Economic Liberty Moral Foundation. Significant indirect (mediation) effects indicated in bold font.

**Table 10:** Mediation effects of moral foundations on the relationship between political orientation and social distancing compliance scale–past (N = 209).

Predictor	*B*	SE	*b* 95% CI [LL, UL]	*t*	*p*	*R* ^*2*^
Constant	7.27	.67	[5.97, 8.58]	10.98	.000	
PO Total Effect	-.44	.06	[-.55, -.32]	-7.49	.000	
Direct Effects						
PO	-.22	.09	[-.39, -.05]	-2.49	.013	
IMF	.25	.15	[-.04, .53]	1.69	.093	
BMF	-.20	.13	[-.45, .05]	-1.56	.121	
ELMF	-.23	.11	[-.45, -.01]	-2.05	.042	
						.21
Indirect Effects						
Total	-.22	.07	[-.35, -.09]			
IMF	-.05	.03	[-.11, .01]			
BMF	-.08	.05	[-.18, .03]			
**ELMF**	**-.10**	**.05**	**[-.20, -.00]**			

Key: PO: Political Orientation; IMF: Individualizing Moral Foundations; BMF: Binding Moral Foundations; ELMF: Economic Liberty Moral Foundation. Significant indirect (mediation) effects indicated in bold font.

**Table 11:** Mediation effects of moral foundations on the relationship between political orientation and social distancing compliance scale–future intended compliance (N = 209).

Predictor	*B*	SE	*b* 95% CI [LL, UL]	*t*	*p*	*R* ^*2*^
Constant	5.86	.94	[4.01, 7.70]	6.26	.000	
PO Total Effect	-.52	.08	[-.68, -.35]	-6.26	.000	
Direct Effects						
PO	-.30	.12	[-.54, -.05]	-2.38	.018	
IMF	.51	.21	[.10, .92]	2.48	.014	
BMF	.00	.18	[-.35, .35]	-.02	.983	
ELMF	-.29	.16	[-.60, .02]	-1.83	.069	
						.14
Indirect Effects						
Total	-.22	.07	[-.35, -.09]			
**IMF**	**-.10**	**.05**	**[-.20, -.01]**			
BMF	-.00	.07	[-.15, .14]			
ELMF	-.12	.07	[-.27, .02]			

Key: PO: Political Orientation; IMF: Individualizing Moral Foundations; BMF: Binding Moral Foundations; ELMF: Economic Liberty Moral Foundation. Significant indirect (mediation) effects indicated in bold font.

**Table 12:** Mediation effects of moral foundations on the relationship between political orientation and social distancing compliance scale–future duration of compliance (N = 209).

Predictor	*B*	SE	*b* 95% CI [LL, UL]	*t*	*p*	*R* ^*2*^
Constant	4.80	.86	[3.11, 6.49]	5.59	.000	
PO Total Effect	-.39	.08	[-.53, -.24]	-5.15	.000	
Direct Effects						
PO	-.20	.11	[-.43, -.03]	-1.73	.086	
IMF	.23	.19	[-.14, .61]	1.24	.216	
BMF	-.04	.16	[-.37, .28]	-.27	.787	
ELMF	-.30	.14	[-.59, -.02]	-2.11	.036	
						.24
Indirect Effects						
Total	-.19	.09	[-.36, -.01]			
IMF	-.05	.04	[-.12, .03]			
BMF	-.02	.07	[-.15, .12]			
ELMF	-.13	.06	[-.25, .00]			

Key: PO: Political Orientation; IMF: Individualizing Moral Foundations; BMF: Binding Moral Foundations; ELMF: Economic Liberty Moral Foundation. Significant indirect (mediation) effects indicated in bold font.

**Table 13:** Mediation effects of moral foundations on the relationship between political orientation and moralization scale (N = 209).

Predictor	*B*	SE	*b* 95% CI [LL, UL]	*t*	*p*	*R* ^*2*^
Constant	4.33	.43	[3.47, 5.17]	10.04	.000	
PO Total Effect	-.35	.04	[-.43, -.28]	-9.29	.000	
Direct Effects					.	
PO	-.24	.06	[-.35, -.12]	-4.12	.000	
IMF	.17	.09	[-.02, .36]	1.81	.071	
BMF	-.01	.08	[-.17, .15]	-.15	.883	
ELMF	-.19	.07	[-.33, -.05]	-2.65	.009	
						.33
Indirect Effects						
Total	-.12	.05	[-.21, -.02]			
IMF	-.03	.02	[-.07, .01]			
BMF	-.00	.03	[-.07, .07]			
**ELMF**	**-.08**	**.03**	**[-.15, -.01]**			

Key: PO: Political Orientation; IMF: Individualizing Moral Foundations; BMF: Binding Moral Foundations; ELMF: Economic Liberty Moral Foundation. Significant indirect (mediation) effects indicated in bold font.

**Table 14:** Mediation effects of moral foundations on the relationship between political orientation and government overreaction scale (N = 209).

Predictor	*B*	SE	*b* 95% CI [LL, UL]	*t*	*p*	*R* ^*2*^
Constant	2.58	.29	[2.01, 3.16]	8.86	.000	
PO Total Effect	.22	.03	[.17, .28]	8.66	.000	
Direct Effects					.	
PO	.15	.04	[.07, .23]	3.87	.000	
IMF	-.08	.06	[-.20, .05]	-1.20	.232	
BMF	-.02	.06	[-.13, .09]	-.43	.671	
ELMF	.16	.05	[.07, .26]	3.35	.001	
						.31
Indirect Effects						
Total	.07	.03	[.01, .14]			
IMF	.01	.04	[-.01, .04]			
BMF	-.01	.02	[-.05, .03]			
**ELMF**	**.07**	**.03**	**[.02, .12]**			

Key: PO: Political Orientation; IMF: Individualizing Moral Foundations; BMF: Binding Moral Foundations; ELMF: Economic Liberty Moral Foundation. Significant indirect (mediation) effects indicated in bold font.

**Table 15:** Mediation effects of moral foundations on the relationship between political orientation health versus economic prioritization scale (N = 209).

Predictor	*b*	SE	*b* 95% CI [LL, UL]	*t*	*p*	*R* ^*2*^
Constant	5.68	.55	[4.58, 6.77]	10.24	.000	
PO Total Effect	.22	.03	[.17, .28]	8.66	.000	
Direct Effects					.	
PO	-.20	.07	[-.35, -.06]	-2.73	.007	
IMF	.50	.12	[.26, .75]	4.12	.0000	
BMF	-.31	.11	[-.52, -.11]	-2.97	.003	
ELMF	-.52	.09	[-.70, -.33]	-5.56	.000	
						.54
Indirect Effects						
Total	-.43	.06	[-.55, .32]			
**IMF**	**-.10**	**.03**	**[-.15, -.05]**			
**BMF**	**-.12**	**.04**	**[-.21, -.04]**			
**ELMF**	**-.22**	**.05**	**[-.31, -.12]**			

Key: PO: Political Orientation; IMF: Individualizing Moral Foundations; BMF: Binding Moral Foundations; ELMF: Economic Liberty Moral Foundation. Significant indirect (mediation) effects indicated in bold font.

Overall, as shown in Tables 9–15, the relationship between political orientation and social distancing outcomes was most often significantly mediated by endorsement of the economic liberty items, which had a significant indirect effect with respect to the social distancing attitudes, past compliance, moralization, government overreaction, and health versus economic prioritization measures. The individualizing foundations significantly mediated the relationship between political orientation and social distancing attitudes, future compliance, and health versus economic prioritization. In contrast, the binding foundations exerted a significant mediation effect only in relation to the health versus economic prioritization measure.

## Discussion

Significant correlations in the predicted directions were obtained between participants’ political orientation and their endorsement of moral foundations. Specifically, there were positive associations between holding a more left-wing political orientation and greater endorsement of individualizing moral foundations and negative associations with both endorsement of binding foundations, and economic liberty items. Political orientation was unrelated to endorsement of lifestyle liberty items. These findings are consistent with previous research [34, 41–43].

Also as predicted, a more left-wing political orientation was positively correlated with participants’ social distancing attitudes, overall self-reported past and future compliance behaviours and intentions, moralization of social distancing compliance and of public health, lower perceptions of government overreaction, and greater endorsement of health versus economic prioritization. These findings also fit with previous research exploring the politicization of short-term social distancing compliance earlier in the pandemic [10, 32, 68], the extent to which social distancing compliance and public health are moralized [47], and wider perceptions of government reactions and priorities [33, 45].

Interestingly, the same significant correlations were generally observed between political orientation, moral foundations, and the outcome variables within both the U.S. and Canadian-based samples. This is noteworthy, given the different party-political landscapes of the two countries, and a further indication of the importance of the left-right spectrum in understanding social distancing responses across these differing national contexts. The notable exception to this was that endorsement of the individualizing foundations was more consistently related to the social distancing outcomes in the U.S. than the Canadian sample. This was not predicted, and future research with larger national samples is thus recommended to test the replicability of these findings.

The hypothesized relations of endorsement of individualizing moral foundations and each of the key outcome measures were found, where endorsement of care and fairness was positively correlated with social distancing attitudes, moralization of social distancing compliance and public health, self-reported compliance behaviours, lower perceptions of government overreaction, and greater endorsement of health versus economic prioritization. These findings are consistent with previous studies linking higher valuation of these individualizing foundations with intended compliance during the early months of the pandemic [10, 30, 49]. The present study extends these findings, by showing that endorsement of these individualizing foundations also relates to other factors surrounding social distancing, including the moralization of violations, and wider perceptions of government reactions and priorities. This makes theoretical sense, as someone who values the individualizing foundations may be more supportive of social distancing measures, from considerations of fairness to others and to reduce the harms posed to them by noncompliance [44]. Moreover, the individualizing foundations are linked to moral emotions including empathy, compassion, and sensitivity to the suffering of others, which may further encourage compliance [30].

In contrast, as predicted, the opposite pattern of significant correlations obtained for endorsement of both the binding and economic liberty items. As the binding foundations place considerable value on the importance of maintaining social connections within groups and institutions [43], they may make the sweeping social distancing restrictions during the pandemic harder to justify [45]. Similarly, those who value economic liberty are also more likely to believe that the negative economic effects of social distancing measures such as lockdowns may outweigh their benefits to public health [33].

Mediation analyses offered some support for the hitherto untested proposition that the relationships between political orientation and many of the key social distancing outcome measures are significantly mediated by endorsement of various moral foundations. Significant effects in the predicted directions were observed for endorsement of both the individualizing and economic liberty foundations regarding attitudes towards social distancing. Valuation of economic liberty was a significant mediator of the relationship between political orientation and self-reported social distancing past compliance, with higher endorsement of economic liberty linked to lower compliance. In contrast, valuation of individualizing foundations was a significant mediator in relation to future intended social distancing compliance. Economic liberty endorsement significantly mediated the relationship between political orientation and moralization of social distancing and public health compliance, with those endorsing economic liberty more highly showing less moralization. Economic liberty also significantly mediated the relationship between political orientation and perceptions of government overreactions in terms of social distancing measures imposed. Moreover, endorsement of individualizing, binding, and economic freedom foundations all significantly mediated the relationship between political orientation and participants’ prioritization of health versus economic concerns, with a positive association observed in relation to individualizing foundations, and a negative link found regarding the other two foundations, as predicted. It is interesting to note that valuation of the economic liberty foundation evidenced the greatest number of significant mediation effects of the relationship between political orientation and the social distancing-related outcomes surveyed, suggesting that it is especially relevant here.

It is also noteworthy that despite the significant bivariate associations found between endorsement of the binding moral foundations and social distancing outcomes, the main and mediation effects observed in relation to these foundations were for the most part nonsignificant. This may reflect the confounding influence of individualizing and economic liberty foundations. It could also reflect the politically somewhat left-leaning nature of the sample, with correspondingly relatively low endorsement of these foundations. Nonetheless, these findings are consistent with the mixed evidence found in previous studies [10, 24]. It could be that the binding foundations offer more ambivalent conclusions than the individualizing ones regarding the wisdom of social distancing. For example, they on the one hand support the need to distance to increase purity and reduce contamination from COVID-19 [10], but on the other emphasize the group ties that social distancing undermines. Additionally, "binders" are sensitive to authority and loyalty, and so are likely to be affected by their leaders’ directives [30]. It could be, then, that the relation between moral foundations and compliance would be moderated by the leaders’ attitudes. Future research is recommended to further probe potential direct and indirect effects of the binding moral foundations on social distancing outcomes.

Overall, the results from the mediation analyses indicate that endorsement of the individualizing and economic freedom moral foundations items can account for a significant amount of the relationship between political orientation and various social distancing attitudes and behaviours. Also worthy of note, though, that political orientation was often the biggest predictor across the final models, and that a portion of the relationship between orientation and social distancing remained unexplained by variations in moral foundations. It is for future research to uncover other potential mediating variables here.

There are likely other ways in which moral foundations influence peoples’ social distancing responses, including the possibility that these influences are also mediated ones. For example, in a study reporting data from 23 countries, found the relationship between moral foundations and a combined measure of prescribed and discretionary COVID-19 preventive behaviours, including social distancing, was mediated by trust in various groups and institutions [69]. Endorsement of individualizing moral foundations was associated with both prescribed and discretionary behavioral intentions via trust in science and, to a lesser extent, via trust in citizens. In contrast, endorsement of the binding moral foundations was indirectly related to these behavioral intentions via trust in government and to a lesser extent via trust in citizens, and had a negative indirect relationship via trust in science. These findings help to elucidate further the relationships between moral foundations and social distancing compliance and invite future research to explore other direct and indirect effects they may exert here.

Understanding political differences in social distancing as partly arising from differences in moral values can increase respectful dialogue between those on different sides of the political spectrum around social distancing, in terms of their different moral priorities regarding protection from harm, versus economic and related freedoms. In turn, there is evidence that when those on the political right or left are presented with reframed arguments that match their underlying moral values, then significant persuasive effects can occur, even on initially polarized issues [27].

Furthermore, findings linking social distancing compliance to differential valuation of the moral foundations may have implications for public health messaging about adherence to social distancing guidelines [10]. Relevant exploratory data, summarized in the supplementary materials section, were gathered in the present study. Overall, a more left-wing political orientation was linked to increased perceived persuasiveness of arguments stressing the individualizing foundations, namely care and fairness, as well as of the fact-based control message. There were no significant correlations between political orientation and the messages stressing the binding or liberty foundations. In contrast, persuasion ratings for each argument condition (except liberty) were significantly related to participants’ scores on the corresponding moral foundations.

Further analyses revealed that across the sample, only the arguments based on the care foundation were rated as more persuasive than the control condition. This is consistent with other research suggesting a low likelihood of short messages outperforming fact-based control messages [63]. This is likely a function of information saturation regarding social distancing and COVID-19 that many people have reached by this point [63]. Also interesting to note is that this finding was observed within both the more left-wing and moderate-to-right wing subsamples. Indeed, arguments stressing the importance of saving lives, especially by protecting people who are vulnerable, are likely to have broad appeal across the political spectrum, given the high valuation of this foundation by those on both sides [34]. This assertion also fits with previous findings showing that other-focused arguments are more persuasive than self-focused ones around public health issues in general [47], and COVID-19 social distancing messaging in particular [63]. Also, those in the more left-wing group rated only those messages based on the individualizing foundations, and the control message, as more persuasive than those in the moderate-right wing group. There were no differences between these groups in the perceived persuasiveness of the arguments based on the binding and liberty foundations.

These exploratory findings warrant replication and more extensive testing. Nonetheless, it is worthy of note that individualizing foundations have featured more than binding and economic liberty ones in public health messaging in general [70], and in COVID-19 messaging specifically [71]. The present findings suggest it could also be useful to offer arguments based around these relatively neglected moral foundations, especially as individuals’ moral foundations were consistently related to their persuasion ratings.

Each paragraph argued for ongoing social distancing compliance, until vaccination rates allowed herd immunity to be achieved. Regarding vaccination, despite the small number in the present sample yet to be offered the vaccine at the time of data collection, there were significant correlations observed between political orientation and COVID-19 vaccine hesitancy, with those on the left less hesitant than those on the right. Furthermore, those who more strongly endorsed the binding and economic liberty foundations were more vaccine hesitant. These findings are consistent with previous work on moral foundations and vaccine hesitancy in general [72] and on the politicization of COVID-19 vaccine uptake [73]. It is for future research to further elucidate these relationships.

### Limitations

The present study comes with several limitations. First, like many studies in this area, an online convenience sample was used. Compared with other data collection modes, online opt-in surveys can yield more honest and accurate self-reports, including less social desirability bias, satisficing, and a lack of interviewer effects [74–76]. They can also yield ethnically and gender diverse samples, as in the present study [77]. However, samples obtained using these methods can differ from nationally representative ones, for example by oversampling younger and more politically left-leaning individuals compared to probability samples [78, 79], as was the case in this study. The generalizability of the current results could thus have been affected by this bias within the sample, either through selection effects among the participants who enrolled in the study or through other unobserved trends that led to the observed political imbalance. Nonetheless, it is noteworthy that the significant relationships observed between political orientation, moral foundations, and social distancing were consistent with previous studies using samples with greater representation from the political right [10, 30]. Another generalizability issue here is that the questionnaire was provided only in English, with no provision in other commonly spoken languages in the two nations, such as French and Spanish. Accordingly, it is recommended that future research further tests the generalizability of current findings.

Speaking further to the somewhat left-leaning nature of the sample in the current study, researchers have noted that political survey responses are sometimes biased and inaccurate, [80–82]. In line with this, a significant majority of Americans (and Canadians) with diverse political views sometimes self‐censor their political opinions, especially those that hold views more on the right-wing of the spectrum [83]. This is particularly likely around issues with large media coverage, like COVID-19. This raises the possibility that the level of right-wing views was underreported in the current sample. Nonetheless, it is noteworthy that a predominantly left-to-centre leaning sample did not impede the uncovering of significant variations in and relationships between political orientations, social distancing, and moral foundations. This is consistent with the more general proposition that the political left is quite heterogenous [84], perhaps even more so than the right [85]. Also worthy of note here is that past studies including more people further to the right have shown similar relationships between politics, moral values, and social distancing [10, 30].

Another important limitation of the present study is that the past and future intended social distancing compliance measures were self-reported. Although commonly used in the literature [30, 49], these are vulnerable to bias. This may be especially so, as the reporting of these behaviours likely has social desirability dimensions [24]. Future research using other methods of sampling social distancing compliance are recommended, for example cell phone data tracking individual mobility [29]. The collection of “other-reported” data would also be useful here [24].

Additionally, there was some conceptual overlap between the SDAS and MFQ scales, as some items in the former measure suggested some reasons to comply or not with social distancing guidelines. Hence, it is unsurprising, for example, that items like "Social distance orders violate my individual rights" related to liberty items in the MFQ, or that responses to "It is our duty as good citizens to follow social distance orders" were associated with endorsement of relevant binding items. Therefore, the degree of content overlap here could have biased the results by inflating the correlation coefficients obtained.

Finally, the current data were collected over a two-month period only (April–June 2021), within a pandemic situation unprecedented in most peoples’ lifetimes. This suggests that the results warrant replication, and that any future studies should account for temporal variations in social distancing guidance, policies, and social norms, notwithstanding the consistency of the current findings with ones obtained from earlier phases of the pandemic [10, 24, 30].

## Conclusion

Despite these caveats, the compatibility of the findings with previous research from earlier stages of the pandemic suggests that the relationships between political, moral, and social distancing variables may have been consistent over time. The lessons learned about how important moral foundations differences are to understanding political differences in peoples’ reactions to social distancing measures during the COVID-19 pandemic could be informative not just in this public health crisis, but in future ones, especially in terms of creating effective messaging to increase compliance whilst balancing health and economic imperatives. It is also hoped that findings such as these may create greater possibilities for mutually empathic dialogues between those with contrasting political attitudes whose actions in times of crisis differ in part due to deep-seated differences in the moral values that they hold most dear.

## Supporting information

S1 File (DOCX)

S2 File (DOCX)

S1 Data (SAV)
