# Supplementary material for: Political orientation, moral foundations, and COVID-19 social distancing
Source: PLoS One. 2022 Jun 24;17(6):e0267136. doi: 10.1371/journal.pone.0267136 (PMC9232135; doi:10.1371/journal.pone.0267136)
Supplement: S1 File — (DOCX) [file pone.0267136.s001.docx]

**Supplementary Information: Measures**

**Demographics Questionnaire**

How old are you? ___________

Which gender do you identify as? ___________

What is your ethnicity? ___________

Which country do you currently live in? ____________

Which state or province do you live in? ____________

Please indicate your highest education level achieved so far: ____________

1 = Less than high school, 2 = High school diploma, 3 = Two-year or associate degree, 4 = Four-year college degree, 5 = Master’s or professional degree, 6 = Doctoral degree.

Please indicate your most recent individual annual income: ____________

Please indicate your most recent household annual income: ____________

Have you ever received a positive test for COVID-19? YES / NO

**Political Orientation Questionnaire**

How would you describe your political views?

Very left-wing 1 2 3 4 5 6 7 Very right-wing

For participants resident in Canada:

Usually, which one of the following federal political parties do you identify most with?

The Conservative Party of Canada

The Bloc Québécois

The Liberal Party of Canada

The New Democratic Party (NDP)

The Green Party of Canada

Other (please specify)

None

On October 21, 2019, there was a federal election in Canada. Were you eligible to vote in that election? YES / NO

If you were eligible, did you vote? YES / NO

If yes, which political party did you vote for?

The Conservative Party of Canada

The Bloc Québécois

The Liberal Party of Canada

The New Democratic Party (NDP)

The Green Party of Canada

Other (please specify)

None

For participants resident in the U.S.:

Usually, which one of the following federal political parties do you identify most with?

Republican

Democrat

Independent

Other (please specify)

None

In November 2020, there was a federal election in the U.S. Were you eligible to vote in that election? YES / NO

If you were eligible, did you vote? YES / NO

If yes, which political party did you vote for in the election for President-Vice President?

Republican

Democrat

Independent

Other (please specify)

**Moral Foundations Questionnaire**

Part 1. When you decide whether something is right or wrong, to what extent are the following considerations relevant to your thinking? Please rate each statement using this scale:

[0] = not at all relevant (This consideration has nothing to do with my judgments of right and wrong)

[1] = not very relevant

[2] = slightly relevant

[3] = somewhat relevant

[4] = very relevant

[5] = extremely relevant (This is one of the most important factors when I judge right and wrong)

______1. Whether or not someone suffered emotionally

______2. Whether or not some people were treated differently than others

______3. Whether or not someone’s action showed love for his or her country

______4. Whether or not someone showed a lack of respect for authority

______5. Whether or not someone violated standards of purity and decency

______6. Whether or not someone was good at math

______7. Whether or not someone cared for someone weak or vulnerable

______8. Whether or not someone acted unfairly

______9. Whether or not someone did something to betray his or her group

______10. Whether or not someone conformed to the traditions of society

______11. Whether or not someone did something disgusting

______12. Whether or not someone was cruel

______13. Whether or not someone was denied his or her rights

______14. Whether or not someone showed a lack of loyalty

______15. Whether or not an action caused chaos or disorder

______16. Whether or not someone acted in a way that God would approve of

______17. Whether or not private property was respected

______18. Whether or not everyone was free to do as they wanted.

Part 2.

Please read the following sentences and indicate your agreement or disagreement:

[0] Strongly disagree

[1] Moderately disagree

[2] Slightly disagree

[3] Slightly agree

[4] Moderately agree

[5] Strongly agree

______19. Compassion for those who are suffering is the most crucial virtue.

______20. When the government makes laws, the number one principle should be ensuring that everyone is treated fairly.

______21. I am proud of my country’s history.

______22. Respect for authority is something all children need to learn.

______23. People should not do things that are disgusting, even if no one is harmed.

______24. It is better to do good than to do bad.

______25. One of the worst things a person could do is hurt a defenseless animal.

______26. Justice is the most important requirement for a society.

______27. People should be loyal to their family members, even when they have done something wrong.

______28. Men and women each have different roles to play in society.

______29. I would call some acts wrong on the grounds that they are unnatural.

______30. It can never be right to kill a human being.

______31. I think it’s morally wrong that rich children inherit a lot of money while poor children inherit nothing.

______32. It is more important to be a team player than to express oneself.

______33. If I were a soldier and disagreed with my commanding officer’s orders, I would obey anyway because that is my duty.

______34. Chastity is an important and valuable virtue.

______35. People who are successful at business have a right to enjoy their wealth as they fit

______36. Society works best when it lets individuals take responsibility for their own lives without telling them what to do.

______37. The government interferes far too much in our everyday lives.

______38. The government should do more to advance the common good, even if that means limiting the freedom and choices of individuals. (Reverse scored)

______39. Property owners should be allowed to develop their land or build their homes in any way they choose, as long as they don't endanger their neighbors.

______40. I think everyone should be free to do as they choose, so long as they don't infringe upon the equal freedom of others.

______41. People should be free to decide what group norms or traditions they themselves want to follow.

**Social Distancing Questionnaires**

**The next set of questions are about social distancing, which** is the practice of reducing physical contact with people outside of the home (e.g., in social, work, or school settings) to avoid transmission of COVID-19 (Coronavirus). Since the start of the COVID-19 virus pandemic, many governments across the world enforced social distancing rules (including keeping a minimum distance from others, bans on large gatherings, stay-at-home measures, and limiting socializing to household members) to slow the spread of the virus.

**Social Distancing Attitudes Scale**

Please indicate your responses to each of the questions below by entering the number that bests describes your opinion.

1: Social distancing has slowed the spread of coronavirus.

Strongly disagree 1 2 3 4 5 Strongly agree

2: Social distancing makes me feel safer.

Strongly disagree 1 2 3 4 5 Strongly agree

3: It is our duty as good citizens to follow social distance orders.

Strongly disagree 1 2 3 4 5 Strongly agree

4: For social distancing to be effective, we need everyone to follow the rules.

Strongly disagree 1 2 3 4 5 Strongly agree

5: Social distancing is not really doing much good.

Strongly disagree 1 2 3 4 5 Strongly agree

6: Social distancing is doing more harm than good.

Strongly disagree 1 2 3 4 5 Strongly agree

7: Social distance orders violate my individual rights.

Strongly disagree 1 2 3 4 5 Strongly agree

8: Social distancing should be a matter of personal choice.

Strongly disagree 1 2 3 4 5 Strongly agree

9: Stopping social distancing too soon will likely lead to another outbreak of coronavirus.

Strongly disagree 1 2 3 4 5 Strongly agree

10: Most places in the country can safely stop social distancing.

Strongly disagree 1 2 3 4 5 Strongly agree

11: We can lift most social distance orders and still keep coronavirus under control.

Strongly disagree 1 2 3 4 5 Strongly agree

12: We need to have better ways to test and track people with coronavirus before we can safely lift social distance orders.

Strongly disagree 1 2 3 4 5 Strongly agree

13: We should not lift social distance orders until there is a steady decline in the number of coronavirus cases.

Strongly disagree 1 2 3 4 5 Strongly agree

14: We should not lift social distance orders if hospitals are full of coronavirus patients.

Strongly disagree 1 2 3 4 5 Strongly agree

**Social Distancing Compliance Scale**

Since the beginning of the COVID-19 pandemic, please indicate how much you have followed each of the following social distancing rules, **when they were in place where you live**, and how long you intend to follow them.

1. Avoiding non-essential gatherings (e.g., social events)

How much have you followed this rule, when it has been in place where you live?

Not at all 1 2 3 4 5 6 7 8 Always

2. Avoiding non-essential travel (domestic, international)

How much have you followed this rule, when it has been in place where you live?

Not at all 1 2 3 4 5 6 7 8 Always

3. Avoiding non-essential trips to stores, restaurants, etc.

How much have you followed this rule, when it has been in place where you live?

Not at all 1 2 3 4 5 6 7 8 Always

4. Avoiding common greetings that involve close contact (e.g., hugs, kisses, handshakes)

Not at all 1 2 3 4 5 6 7 8 Always

5. Avoiding socializing in person with family members who do not typically live with you

How much have you followed this rule, when it has been in place where you live?

Not at all 1 2 3 4 5 6 7 8 Always

6. Avoiding socializing in person with close friends

How much have you followed this rule, when it has been in place where you live?

Not at all 1 2 3 4 5 6 7 8 Always

7. Avoiding or limiting contact with people at high risk or vulnerable populations (for example other adults, those with at risk conditions and those in poor health)

How much have you followed this rule, when it has been in place where you live?

Not at all 1 2 3 4 5 6 7 8 Always

8. Keeping the recommended safe distance from people who do not typically live with you

How much have you followed this rule, when it has been in place where you live?

Not at all 1 2 3 4 5 6 7 8 Always

9. Overall, how much have you followed social distancing rules in general?

Not at all 1 2 3 4 5 6 7 8 Always

10. From now on, how much do you intend to follow social distancing rules in general?

Not at all 1 2 3 4 5 6 7 8 Always

How much longer would you be willing to follow some form of social distancing rules from now? < 1 month / 1-3 months / 4-6 months / 7-9 months / 10-12 months / > 12 months

**Moralization Scale**

To what extent do you feel that violating social distancing rules is morally condemnable (i.e., how "wrong" is such behavior)?

1 Totally acceptable to violate social distancing rules

2

3

4

5 Totally unacceptable to violate social distancing rules

To what extent do you think that public health is a moral issue?

Not at all 1 2 3 4 5 Extremely

**Government Overreaction to COVID-19 Scale**

Governments around the world have also imposed many different types of restrictions at various points during the COVID-19 pandemic. Please indicate how much you feel each of the following restrictions has been an underreaction vs. overreaction to the pandemic.

1. Restrictions on social contacts outside of household members

How appropriate do you think that this response has been to COVID-19?

1 = Significant Underreaction

2 = Slight Underreaction

3 = Appropriate Reaction

4 = Slight Overreaction

5 = Significant Overreaction

2. Travel restrictions (domestic and international)

How appropriate do you think that this response has been to COVID-19?

1 = Significant Underreaction

2 = Slight Underreaction

3 = Appropriate Reaction

4 = Slight Overreaction

5 = Significant Overreaction

3. Closing of cultural and sporting events and facilities

1 = Significant Underreaction

2 = Slight Underreaction

3 = Appropriate Reaction

4 = Slight Overreaction

5 = Significant Overreaction

4. Closing of schools and universities

1 = Significant Underreaction

2 = Slight Underreaction

3 = Appropriate Reaction

4 = Slight Overreaction

5 = Significant Overreaction

5. Closing of non-essential businesses (e.g., gyms, restaurants etc.)

1 = Significant Underreaction

2 = Slight Underreaction

3 = Appropriate Reaction

4 = Slight Overreaction

5 = Significant Overreaction

6. Imposing of curfews

1 = Significant Underreaction

2 = Slight Underreaction

3 = Appropriate Reaction

4 = Slight Overreaction

5 = Significant Overreaction

7. Restrictions on non-essential trips to stores and restaurants

1 = Significant Underreaction

2 = Slight Underreaction

3 = Appropriate Reaction

4 = Slight Overreaction

5 = Significant Overreaction

8. Limits on visitations to hospitals and care homes

1 = Significant Underreaction

2 = Slight Underreaction

3 = Appropriate Reaction

4 = Slight Overreaction

5 = Significant Overreaction

**Attitudes towards Health vs Economy Prioritization Scale**

During the pandemic, governments have had to manage both the health and economic impacts of the pandemic. Please read through each of the statements below about these impacts and indicate your level of agreement or disagreement with each one.

At this stage of the pandemic, I am more concerned about the economic impact of the COVID-19 outbreak than the public health impact

1 – Very strongly disagree 2 – Strongly disagree 3 – Disagree 4 – Neither agree nor disagree 5 – Agree 6 – Strongly agree 7 – Very strongly agree

At this stage of the pandemic, ongoing social distancing needs to stay in place to slow the spread of COVID-19, even though this will continue to hurt the economy

1 – Very strongly disagree 2 – Strongly disagree 3 – Disagree 4 – Neither agree nor disagree 5 – Agree 6 – Strongly agree 7 – Very strongly agree

At this stage of the pandemic, the highest government priority should be to save as many lives as possible, despite this slowing the economic recovery. 1 – Very strongly disagree 5 – Very strongly agree

1 – Very strongly disagree 2 – Strongly disagree 3 – Disagree 4 – Neither agree nor disagree 5 – Agree 6 – Strongly agree 7 – Very strongly agree

At this stage of the pandemic, it is more of a priority for the government to protect jobs and restart the economy than to take every last precaution against COVID-19.

1 – Very strongly disagree 2 – Strongly disagree 3 – Disagree 4 – Neither agree nor disagree 5 – Agree 6 – Strongly agree 7 – Very strongly agree

**Vaccine Hesitancy**

The next few questions ask about your opinions about COVID-19 vaccination. Please indicate your response to each of the questions below.

1. Have you had the opportunity to receive a COVID-19 vaccination yet? YES / NO

2. If yes, did you choose to get the vaccination or to refuse it? YES, GOT THE VACCINATION / NO, REFUSED THE VACCINATION.

**Oxford Covid-19 Vaccine Hesitancy Scale**

Would you take a COVID-19 vaccine if offered?

1 = Definitely

2 = probably

3 = I may or may not

4 = Probably not

5 = Definitely not

Don’t know

When a COVID-19 vaccine is available for me to take:

1 = I will want to take it as soon as possible

2 = I will take it when offered

3 = I’m not sure what I will do

4 = I will put off (delay) getting it

5 = I will refuse to get it

Don’t know

I would describe my attitude towards receiving a COVID-19 vaccine as:

1 = Very keen

2 = Pretty positive

3 = Fairly neutral

4 = Quite uneasy

5 = Against it

Don’t know

If a COVID-19 vaccine was available at my local pharmacy, I would:

1 = Get it as soon as possible

2 = Get it when I have time

3 = Delay getting it

4 = Avoid getting it for as long as possible

5 = Never get it

Don’t know

If my family or friends were thinking of getting a COVID-19 vaccine, I would:

1 = Strongly encourage them

2 = Encourage them

3 = Not say anything to them about it

4 = Ask them to delay getting a vaccine

5 = Suggest that they do not get the vaccine

Don’t know

I would describe myself as:

1 = Eager to get a COVID-19 vaccine

2 = Willing to get a COVID-19 vaccine

3 = Not bothered about getting a COVID-19 vaccine

4 = Unwilling to get a COVID-19 vaccine

5 = Anti-vaccination for COVID-19

Don’t know.

Taking a COVID-19 vaccine is:

1 = really important

2 = important

3 = neither important nor unimportant

4 = unimportant

5 = really unimportant

Don’t know.

**Moral Arguments (order of presentation randomized)**

Despite the fatigue that many people are experiencing in continuing to follow social distancing rules, a number of experts think that they will need to stay in place for a significant amount of time to come, until the majority of people have been vaccinated, which will mean that herd immunity has been reached. There are lots of different arguments being made about why this is important.

Please read through each of the short paragraphs below, which are different types of arguments why continuing to follow social distancing rules until then is important and enter your responses to the questions about each one. Try to evaluate how persuasive you think that each argument is, separately from the other paragraphs.

(Care/harm): Following social distance rules until most people have been vaccinated and herd immunity is reached is important because:

We need to keep as many people safe from the virus as possible until enough people have had the vaccine. By following the rules, we are showing compassion for those who are most vulnerable. We need to protect the sick, the elderly, and immuno-compromised, especially those who cannot get the vaccine, or for whom it will not be effective. By doing these things, we are caring for those most likely to be harmed by the virus, and reducing the suffering caused.

How persuasive do you think this message is?

Not at all 1 2 3 4 5 Extremely

How convincing do you think this message is?

Not at all 1 2 3 4 5 Extremely

How much do you think this message made its point?

Not at all 1 2 3 4 5 Extremely

(Fairness/reciprocity): Following social distance rules until most people have been vaccinated and herd immunity is reached is important because:

The virus impacts minority groups, and people living in poverty, disproportionately. If we all continue to follow the guidelines until enough people have had the vaccine, then we are being fair to everyone, as we all have an equal right to be protected from the virus as much as possible. Only by doing this can we reduce its inequitable impacts. Doing these things is a matter of social justice.

How persuasive do you think this message is?

Not at all 1 2 3 4 5 Extremely

How convincing do you think this message is?

Not at all 1 2 3 4 5 Extremely

How much do you think this message made its point?

Not at all 1 2 3 4 5 Extremely

(Liberty/oppression): Following social distance rules until most people have been vaccinated and herd immunity is reached is important because:

Individuals have a right not to be infected by others, and so we all need to choose to follow the guidelines. These guidelines are the best way to return individuals to liberty, with the freedom to do what they want. By continuing to follow the guidelines until enough people have had the vaccine, individuals can get back to taking responsibility for their own lives. If we don’t do this, then the government will continue to tell us what to do.

How persuasive do you think this message is?

Not at all 1 2 3 4 5 Extremely

How convincing do you think this message is?

Not at all 1 2 3 4 5 Extremely

How much do you think this message made its point?

Not at all 1 2 3 4 5 Extremely

(Authority/respect): Following social distance rules until most people have been vaccinated and herd immunity is reached is important because:

We have a duty to obey public health officials and politicians when they tell us we must slow the spread of coronavirus. It is critical to continue to defer to the authority of our leaders during this crisis. Complying with the rules is needed until enough people have had the vaccine. Following our leaders is the best way to serve the greater cause here.

How persuasive do you think this message is?

Not at all 1 2 3 4 5 Extremely

How convincing do you think this message is?

Not at all 1 2 3 4 5 Extremely

How much do you think this message made its point?

Not at all 1 2 3 4 5 Extremely

(Control): Following social distance rules until most people have been vaccinated and herd immunity is reached is important because:

Coronavirus is a respiratory illness that can lead to severe symptoms, and even be fatal. It can spread quickly though close personal contact. We can help prevent the spread of COVID-19 by following social distancing guidelines. We all need to continue to do this until enough people have had the vaccine.

How persuasive do you think this message is?

Not at all 1 2 3 4 5 Extremely

How convincing do you think this message is?

Not at all 1 2 3 4 5 Extremely

How much do you think this message made its point?

Not at all 1 2 3 4 5 Extremely

(Purity/sanctity): Following social distance rules until most people have been vaccinated and herd immunity is reached is important because:

The effects of COVID-19 are disgusting, threatening our bodies by damaging tissue

and impairing our breathing. Until enough people are vaccinated, this contagious virus will continue to spread. We need to continue to follow the rules to keep our bodies as pure and our communities as clean as possible from this repulsive virus! It is the virtuous thing to do and shows our integrity.

How persuasive do you think this message is?

Not at all 1 2 3 4 5 Extremely

How convincing do you think this message is?

Not at all 1 2 3 4 5 Extremely

How much do you think this message made its point?

Not at all 1 2 3 4 5 Extremely

(Loyalty/ingroup): Following social distance rules until most people have been vaccinated and herd immunity is reached is important because:

We all need to do this for our families, friends, and fellow citizens. Continuing to follow these guidelines is the most patriotic thing we can do to help our communities and country. Coronavirus has invaded our nation and we need be loyal in our fight against it. Only by staying united until enough people have been vaccinated can we defeat this deadly enemy.

How persuasive do you think this message is?

Not at all 1 2 3 4 5 Extremely

How convincing do you think this message is?

Not at all 1 2 3 4 5 Extremely

How much do you think this message made its point?

Not at all 1 2 3 4 5 Extremely
