# Supplementary material for: Political orientation, moral foundations, and COVID-19 social distancing
Source: PLoS One. 2022 Jun 24;17(6):e0267136. doi: 10.1371/journal.pone.0267136 (PMC9232135; doi:10.1371/journal.pone.0267136)
Supplement: S2 File — (DOCX) [file pone.0267136.s002.docx]

**Supplementary Materials**

**Persuasion analyses**

To test the relationship between participants’ political orientation, moral foundations, and how persuasive they found messages supporting ongoing social distancing compliance, Pearson product-moment correlations were calculated among participants’ persuasion ratings for each of the 7 argument conditions, their respective moral foundation scores, and political orientation. The significance of the observed correlations was then determined (see Table 16 below).

**Table 16 - Correlations between Persuasion Ratings, Political Orientation, and Moral Foundations (n=209)**

| Argument Condition | Political Orientation | Relevant Moral Foundation Score |
| --- | --- | --- |
| Harm | -.47** | .33** |
| Fairness | -.57** | .44** |
| Authority | -.03 | .25** |
| Loyalty | -.04 | .22** |
| Purity | -.04 | .25** |
| Liberty | -.11 | -.01 |
| Control | -.26** | N/A |

** p < .001 (two-tailed).

As shown in Table 16, political orientation was significantly correlated with only the persuasion ratings for the harm, fairness, and control conditions, with those more on the right less persuaded by each of these. In contrast, there were more consistent significant correlations between these persuasion ratings and all moral foundations, except liberty. In other words, moral persuasion ratings were more consistently related to participants’ moral values than to their political orientation.

To explore differences between persuasion ratings across conditions, a repeated measures ANOVA was conducted. Mauchly’s test indicated that the assumption of sphericity had been violated, *x2* (20) = 94.58, p<.001, therefore degrees of freedom were calculated using the Greenhouse-Geiser estimates of sphericity (e = .87). There was a significant effect of argument condition, *F* = (1, 5.190) = 109.07, *p* = <.001). The outcomes of Tukey’s HSD post hoc testing with Bonferroni correction revealing significant pairwise differences between the argument conditions are displayed in Table 17 below.

**Table 17 - Means and Standard Deviations of Persuasiveness by Condition**

| Argument Condition | *M* | *SD* |
| --- | --- | --- |
| Care/harm | 3.92a | .97 |
| Control | 3.44b | 1.09 |
| Fairness/cheating | 3.13c | 1.13 |
| Liberty/oppression | 3.00cd | 1.14 |
| Loyalty/betrayal | 2.84d | 1.16 |
| Purity/degradation | 2.21e | 1.07 |
| Authority/subversion | 2.16e | 1.13 |

Key: Means that do not share subscripts differ by p < .05 according to Tukey’s Honestly Significant Difference.

As depicted in Table 17, the arguments in the care/harm condition were rated as significantly more persuasive than all other conditions, and this was the only condition rated as more persuasive than the factual control condition. The sample was then divided into two groups, the first those participants with a left-wing orientation (N = 144), and the second those with moderate or right-wing orientations (N = 65). Once again, the care/harm message was the only one rated as more persuasive than the factual control message in both groups.
